# Amplifying perceptual demands: How changes in the colour vests affect youth players performance during medium-sided games

**DOI:** 10.1371/journal.pone.0262245

**Published:** 2022-01-14

**Authors:** Diogo Coutinho, Bruno Gonçalves, Hugo Folgado, Bruno Travassos, Sara Santos, Jaime Sampaio

**Affiliations:** 1 Department of Sports Sciences, Exercise and Health, University of Trás-os-Montes and Alto Douro, Vila Real, Portugal; 2 Research Center in Sports Sciences, Health Sciences and Human Development, CIDESD, CreativeLab Research Community, Vila Real, Portugal; 3 University Institute of Maia, ISMAI, Maia, Portugal; 4 Departamento de Desporto e Saúde, Escola de Saúde e Desenvolvimento Humano, Universidade de Évora, Évora, Portugal; 5 Comprehensive Health Research Centre (CHRC), Universidade de Évora, Évora, Portugal; 6 Portugal Football School, Portuguese Football Federation, Oeiras, Portugal; 7 Department of Sports Sciences, University of Beira Interior, Covilhã, Portugal; Manchester Metropolitan University - Cheshire Campus, UNITED KINGDOM

## Abstract

This study explored how manipulating the colour of training vests affects footballers’ individual and collective performance during a Gk+6vs6+Gk medium-sided game. A total of 21 under-17 years old players were involved in three experimental conditions in a random order for a total of four days: i) CONTROL, two teams using two different colour vests; ii) SAME, both teams wearing blue vests; iii) MIXED, all 6 players per team wore different colour vests. Players’ positional data was used to compute time-motion and tactical-related variables, while video analysis was used to collect technical variables. Further, these variables were synchronized with spatiotemporal data allowing to capture ball-related actions in a horizontal 2D plan. All variables were analysed from the offensive and defensive perspective. From the offensive perspective, players performed more and further shots to goal during the CONTROL than in SAME and MIXED (small effects) conditions, with a decreased distance to the nearest defender (small effects). While defending, results revealed lower distance to the nearest teammate (small effects) in the CONTROL than in the SAME and MIXED conditions, and higher team longitudinal synchronization (small effects). In addition, the CONTROL showed in general lower values of team width while defending than in the other 2 conditions. Overall, coaches may use the CONTROL condition to emphasize offensive performance and defensive behaviour over the longitudinal direction with increased physical demands. In turn, coaches may use the manipulation of players vests to emphasize defensive performance, as players seem to behave more cohesively under such scenarios.

## Introduction

In association football, decision-making has been linked with the players’ ability to interact with surrounding information to unfold goal-directed behaviour [1, 2]. Examples of these sources of information are the teammates’ movements [3], the opponents positioning [4], the ball [5] and spatial references such as the goal location [6] or field external lines [2]. From the interaction with these sources of information, opportunities for action (i.e., affordances) will emerge and will guide the players’ movement behaviour [7, 8]. However, this information is dynamically changing, and new opportunities for action are continuously arising according to changes in-game environment [9]. From a practical perspective and as an example, the exploratory behaviour of a midfielder before receiving the ball should identify the spatial-temporal tendencies of opponents in relation to forward or win players that sustain a decision of an interior penetrative pass, a cross to the corridors, or other action that concurs to the group common goal.

These implications have a major impact on the design of training tasks, which should challenge the players to continuously couple their actions to the information available on the surrounding environment [10]. Under this scope, the constraint-led approach emphasizes the role of constraints manipulation to guide players movement behaviour [11, 12]. For that purpose, coaches intentionally adjust key individual and tasks constraints to highlight specific information that will shape players movement patterns [10, 12, 13]. From this perspective, small to large -sided games (SSG, small-sided games; MSG, medium-sided games; LSG, large-sided games) have been used to develop players’ decision-making, as it allows the players to couple their actions to the specifying information (i.e., relevant to support goal-directed behaviours) as result of manipulations in its rules [11]. In addition, SSG, MSG and LSG are able to represent the inherent variability found in competitive performance environments, helping players to cope with these dynamic contexts [8, 10, 14].

That is, coaches can manipulate the game rules with the intention of guide the players’ movement behaviour as a result of the perception of the available information [8, 11]. During the last decade, there has been a growing body of research exploring how different constraints affected the players positional, technical and physical responses during SSG, MSG and LSG. For example, it was shown that increase the pitch width during a Gk+4vs4+Gk SSG encourage the players to adopt wider positions, contributing to a higher number of lateral passes and shots-on-target [6]. The impact of adding corridor and sectorial lines during a Gk+6vs6+GK MSG was also inspected. In this respect, it was found that the lines emphasize information related to the distance, promoting higher team dispersion, spatial regularity and distance covered at high intensity, possibly as result of the lower movement synchronization [15]. Players’ movement synchronization and external load seems also to be affected by the game rules, as playing in restricted spaces during a GK+10vs9 LSG seems to limit the players movement synchronization and distance covered [16]. These studies have been helping coaches in the design of informational-relevant training tasks [17]. However, and despite the great contributes that resulted from those works, there is still a lack of research exploring how different perceptual manipulations (e.g., varying the players vests by having both teams wearing the same colour) impacts the players’ physical, technical and tactical behaviour.

Taking into consideration the way how the environment shapes the players’ decisions, coaches should design training tasks that amplify players’ perceptual demands to improve action capabilities [18]. Indeed, past research has shown that expert players decide faster and better than non-expert players, which has been linked in the way how they explore the specifying information [19, 20]. For example, research surrounding the scanning, which is a term referring to visual exploratory head movements performed by the player in the moments prior to receiving the ball, has shown that players with a higher scanning frequency are more likely to act faster and perform successful passes [21–23]. As so, training tasks may not only represent the same information found in competitive environments, but also challenge the players to explore their surrounding more often to successfully perform. Under this scope, game-based training tasks are usually performed while confronting two teams that are well defined (e.g., one team wearing red vests while the other team wearing green vests). However, using different vests per team may decrease the need that the players must explore the surrounding, as it may be easier, using their peripheral vision, to perceive the teammates and opponents’ positions. In turn, exposing the players to conditions where it may be harder to identify the teammates and opponents’ (e.g., both teams wearing the same outfit or each player from each team using a different colour vest), and consequently lead to different movement behaviours as a result of the change in perceptual demands. In fact, previous reports have shown that the colour of players outfit seems to affect their performance [24, 25]. For example, when considering the chances of winning at home in the English Premier league, Attrill, Gresty [24] found that the teams wearing red had a higher percentage of points achieved and higher mean position on the table compared other colours. In addition, it was shown that the colour outfit affects players’ spatial perception, mainly their ability to properly identify the teammates positioning [25]. These effects may be related with the mental time to process the colours, as is seems that some colours (e.g., yellow) increases the visual choice reaction time than others (e.g., green or red) [26]. More recently, it was added that players take longer to discriminate players from both teams when wearing crossed outfits compared to uniform (i.e., using only one colour), while also some confusion might emerge when players’ are using the same-coloured shorts [27]. In contrast to these studies, one laboratory-based study questioned players to decide to whom to pass and to recall information presented in a 6m concave immersive screen in which players’ jersey colour was varied [28]. Overall, the study showed that the visual angle affected the decision-making, but not the colour of the jerseys [28]. Despite the important contributes from the previous study, no study to date has addressed how players positioning behaviour may be affected by varying the colour of the teammates and opponents’ outfits.

In addition, while the ball is a key element that constraint the way that players explore the surrounding environment [5], research including analysis with the ball, particularly during training drills, is still very scarce. Under this scope, Folgado et al. [6] developed a new method to track the ball positioning and integrate it with the players relative positioning, which may provide additional insights regarding players behavioural dynamics when in interaction with the surrounding information. Therefore, further research exploring how players’ adjust their positioning in relation to the ball location and the manipulation of specific task constraints is welcome. Thus, this study aimed to explore how manipulations in youth players (under-17, U17) vests differences between teams affected their physical, individual and collective tactical performance during a Gk+6vs6+Gk MSG. Under this perspective, it was hypothesized that players would perform more offensive actions while showing higher values of synchronization when playing with different colour vests (i.e., as it happens during competitive performances). In contrast, it is expected that players use more space, stay more on the ball and run more when wearing with the same colour (i.e., blue vs blue) or with several colours (i.e., each player from each team wearing a different colour vest) as result of a possible increase in the perceptual demands.

## Methods

### Participants

Twenty-one youth football players from the same club participated in this study (age = 16.2 ± 0.6 years; Height = 173.1 ± 8.4 cm; Weight = 65.3 ± 6.6 kg; Years of experience = 8.3 ± 2.8 years). Power analysis was first performed to determine the required sample size. The sample size was calculated with G*Power (Version 3.1.5.1 Institut für Experimentelle Psychologie, Düsseldorf, Germany) for an effect size of 0.6, an α of 0.05, and a power of 0.8 (1–β) [29]. The total sample sized computed with this method was a minimum of 19 players. All players were engaged in four training sessions per week (90 to 105 minutes per session) and had an official eleven-a-side match during the weekend. At the time of the study, there were no injured players, and therefore, all participated in the study. Goalkeepers were part of the study but were excluded from the data analysis, since their positioning is very restricted to a specific field area and their game dynamics are different from the outfield players. A written and informed consent was provided to the coaches, players, and by their legal guardians, as well as by the club, before the beginning of the study. All participants were notified that they could withdraw from the study at any time. The study protocol followed the guidelines and was approved by the local Ethics Committee of the Research Center in Sports Sciences, Health Sciences and Human Development (UIDB/4045/2020) and conformed to the recommendations of the Declaration of Helsinki.

### Study design

The study design was based on a repeated-measures approach under 3 experimental conditions: (i) team A playing with a green vest while team B playing with red vest (CONTROL); (ii) both team A and B playing with same blue t-shirt (SAME); and (iii) every player from each team had a different colour vest (red, blue, light blue, green, orange, white—MIXED). For this purpose, the games were played using a Gk+6vs6+Gk, as it has been shown to provide a similar team structure as those found in the 11-a-side competitive match (1 Goalkeeper, 2 players in the back, 3 players occupying the three channels and 1 striker).

### Procedures

All players were tested during five sessions over three weeks during the middle of the in-season competitive period (season 2017/2018). The first session (performed in the first week), was used to familiarize the players to the MSG conditions, by playing one bout of 6-min for each condition, with a 3-min passive recovery within the bouts. Only one session using the same procedures (Gk+6vs6+Gk in the same space) was performed as players often performed training drills adopting the same conditions as those found in the present study. A total of two MSG (2x 3 bouts of 6-min) were performed to ensure that all players experienced the experimental conditions. Then, the remaining four sessions were used to test the experimental conditions. In all testing days, the session started at the same time of the day, to avoid the effects of circadian rhythms on the results, and over similar weather conditions. Before the experimental conditions, the players performed a 15-min warm-up based on low-intensity running and a 6-a-side ball possession game.

### Experimental task

For each testing day, the head coach selected twelve players (plus two goalkeepers) that allowed to divide the group into two balanced teams, taking into account the coach perception of the players physical, technical, tactical and perceptual skills [30]. In addition, the players’ playing position was also considered as the head coach selected 2 centre backs (n = 4), 2 wide midfielders / fullbacks (fullbacks n = 4, wide midfielders n = 5), 1 midfielder (n = 4) and 1 striker (n = 3) to each team. The teams were kept constant during the session, where the players were randomly exposed to all the three experimental conditions, but varied from day to day to ensure a higher data generalization (i.e., one fullback may be assigned to participate in the session #2 and perform the three conditions, but not participate in the following session #3). Each day players performed three bouts of 6-min interspersed with a 3-min passive recovery in between as it consisted in the usual time adopted by the coach during regular practices [31], where each bout consisted in one of the three conditions (CONTROL, SAME, MIXED) played in random order. In addition, these time periods have been consistently used in game-based situations attempting to analyse the players’ movement behaviour [6, 14, 15]. The game consisted of a Gk+6vs6+Gk S MSG SG on a 64x43m artificial turf field (length x width; ~200m^2^ relative playing space per player), as it is one of the most common playing formats adopted in youth football [32]. In addition, playing formats with more players (i.e., above the 4vs4) seems to be more appropriate to capture positioning dynamics [33]. This Field was an official 7-a-side field, and so all field lines were properly marked on the floor with white ink. Accordingly, past research has shown that the type of field external markings interfere with youth players positional, physical and technical performance [2]. Several balls were placed around the field to ensure its replacement as fast as possible, decreasing the time that the ball was out of play. No coach feedback or encouragement was allowed during the conditions. The players were encouraged to hydrate with water before the MSG and also in-between the bouts. Apart from the off-side rule that was not applied and the restart of the game by the goalkeeper that conceded a goal to ensure a fast restart, all the remaining rules were played according to the FIFA football rules.

### Data collection

Positional data and the distance covered during MSG were gathered using 5 Hz global positioning system (GPS) units (SPI-PRO, GPSports, Canberra, ACT, Australia). The players’ latitude and longitude information obtained with the GPS units were resampled to remove possible data gaps and to synchronize all the individual data. Following this procedure, the data were converted to meters using the Universal Transverse Mercator (UTM) coordinate system together with a rotational matrix to adjust the players displacement data, field length and width with the appropriate x and y-axis. This procedure was carried out by the data retrieved from four GPS units placed on each field corner [34]. In addition, all game-based scenarios were recorded using a digital video camera, Sony NV-GS230, that was fixed at a 2-m height and aligned in the midfield part of the field. In addition, and prior to the start of each game-based bouts, a GPS enabled watch allowed to identify the right time in which each bout started, allowing to synchronize the data between the GPS with the video file [6]. The video files were downloaded to a computer and notational analysis software (Longomatch, version 1.3.7., Fluendo) was used to register the time of every ball-related action during the game-based conditions, following existing data processing procedures [6]. The following actions were considered: player gaining ball possession, player disposing the ball possession; player touching the ball without gaining possession; ball over the side line; ball over the end line; ball hitting the crossbar/post; ball shooting; goal scoring and fouls. The position of the ball in the 2D horizontal plane was modelled according to an algorithm that integrate the players relative positioning collected by the GPS system and the notational data resulting from the video analysis, synchronising the events with the GPS time. For this purpose, the ball was represented by the same position as the player in possession and it was considered that it followed a straight patch between the starting and ending position when passed or shot. In addition, to recreate other non-GPS ball specific positions, such as the goal, end and side lines, a total of 18 fixed locations were defined according to the field referential [for details please see 6].

The videos were analysed by an experienced performance analyst, and the data reliability was inspected by retesting 20% of the sample. The intraclass correlation was deemed as high (>0.89) [35]. In addition, the original video from each MSG scenario was also visually compared to the correspondent 2D representation, and any possible conflicting situation was corrected prior to the data analysis.

### Physical variables

The total distance covered (per minute), the distance covered at different movement speed categories (per minute) for each player were also calculated [16]. The following speed categories were considered for analysis: walking (0.0–3.5 km/h); jogging (3.6–14.3 km/h), running (14.4–19.8 km/h) and sprinting (> 19.9 km/h) [16]. Based on the ball possession, both the positional and physical-related variables were analysed according to the offensive and defensive phase of the MSG.

### Individual tactical variables

The individual tactical variables were: (a) number of forward, lateral and backward completed passes; (b) the number of dribbles; (c) total distance covered with the ball while dribbling; (d) number of shots; and (e) distance to the nearest defender during the shot [6]. For the passing direction classification, it was considered the following angles in relation to the centre of the field and second quadrant: forward passes between 0° and 45°; lateral passes between 45° and 135°; and backward passes between 135° and 180°.

### Collective tactical variables

The positional data of the players were used to calculate the intra-team coordination tendencies based on the time that players’ dyads spent synchronized in both longitudinal and lateral directions. These variables were calculated with the relative phase and the Hilbert transform [36]. The movement synchronization of each dyad was quantified by the percentage of time spent between -30° to 30° bin (near-in-phase mode of coordination) [34]. Also, data was used to assess the distance from each player to: (i) the nearest teammate and (ii) nearest opponent, expressed by the absolute values (m) and the coefficient of variation (CV). Finally, the team length (m) and width (m) were also inspected.

### Statistical analysis

The data were presented as means (M) ± standard deviations (SD). All data were assessed for outliers and assumptions of normality. Due to the existence of normal and non-normal distribution of data, the differences between conditions were assessed either using parametric and non-parametric tests (ANOVA and Kruskal-Wallis, respectively) for each game scenario. Statistical significance was set at p < .05 and calculations were carried out using SPSS software V24.0 (IBM SPSS Statistics for Windows, Armonk, NY: IBM Corp.).

Complementary, pairwise differences (CONTROL vs SAME; CONTROL vs MIXED) were assess via differences in group means expressed in raw data units with 90% confidence limits (CL). Thresholds for effect size statistics were: <0.2, trivial; <0.6, small; <1.20, moderate; <2.0, large; and >2.0, very large [37].

## Results

### Offensive phase

**Fig 1:**
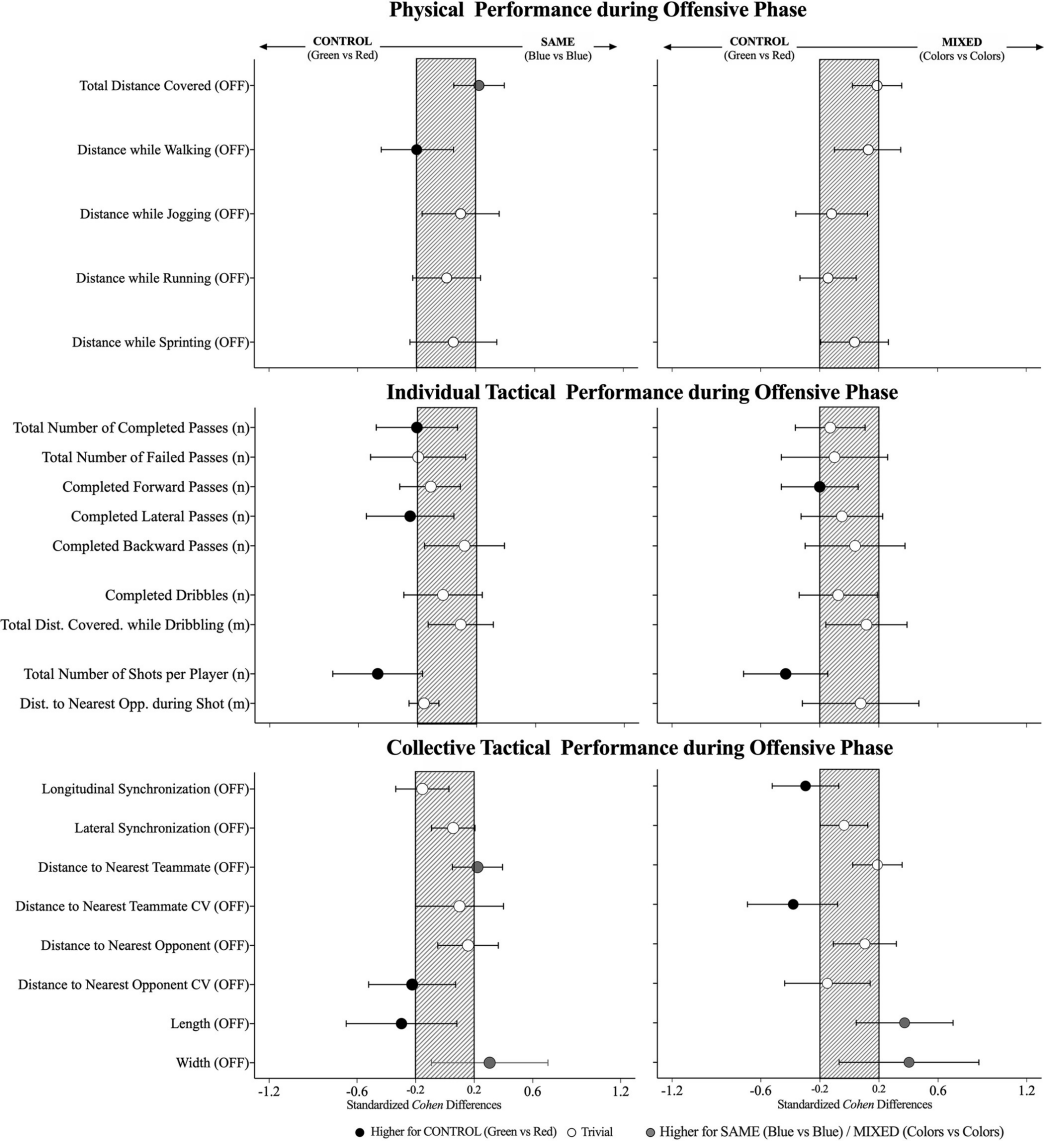
Standardised (Cohen) differences in physical, individual and collective tactical variables according to the type of vest (CONTROL, SAME and MIXED) during the offensive phase. Error bars indicate uncertainty in the true mean changes with 90% confidence intervals. OFF = offensive.

The effects of manipulating the colour of training vests are presented in Table 1 and Fig 1. The results from the individual tactical performance showed significant effects in the number of total shots. Accordingly, there was a small decrease in the number of shots (CONTROL vs SAME: -0.25, ±0.16; raw mean differences, ±90 CL; CONTROL vs MIXED: -0.23, ±0.15; X^2^ = 7.63, *P* **≤** 0.05) during SAME and MIXED compared to the CONTROL. In addition, some trends were identified in the passing action, such as a higher number of lateral passes in CONTROL compared to SAME (small effects) and higher number of forward passes in CONTROL comparatively to MIXED (small effects). Results from the collective tactical performance showed a small increase in distance to the nearest teammate (0.30; ±0.23, *F* = 2.49, *P* **≤** 0.05), from the CONTROL to the SAME. There were small higher values (~4 more) in the longitudinal synchronization in the CONTROL compared to the MIXED, while also small higher values (~1.2 more) were identified in the CV from the distance to the nearest teammates when comparing the CONTROL with the MIXED and in the CV in the distance to the nearest opponent (~1.7 more) in the CONTROL compared with the SAME. Finally, higher length values were found (~1.3 more, small effects) during the CONTROL compared to the SAME, while lower values (~1.6 lower, small effects) were found when compared to the MIXED. In addition, the results showed small lower width values in the CONTROL than in the other conditions.

**Table 1:** Descriptive and statistical analysis for physical, individual and collective tactical-related variables according to the type of vest used during the offensive phase.

Variables	Game-Based Conditions			Difference in means (±90% Confidence limits)		
	CONTROL	SAME	MIXED	CONTROL vs SAME	CONTROL vs MIXED	*P*
	(Green vs Red)	(Blue vs Blue)	(Colours vs Colours)			
	(Mean±SD)—(%)	(Mean±SD)—(%)	(Mean±SD)—(%)			
**Offensive Physical Variables**						
Total Distance Covered (m)	7.40±1.09	7.70±1.47	7.65±1.34	0.30; ±0.23	0.25; ±0.22	.087
Distance Covered while Walking (m)	6.54±2.38	6.07±2.26	6.85±2.53	-0.48; ±0.59	0.31; ±0.55	.109
Distance Covered while Jogging (m)	96.76±13.19	98.00±10.73	95.25±13.15	1.25; ±3.31	-1.50; ±3.08	.368
Distance Covered while Running (m)	20.06±10.22	20.10±15.98	18.13±12.69	0.05; ±3.10	-1.92; ±2.57	.185
Distance Covered while Sprinting (m)	4.23±4.53	4.47±5.07	4.41±4.75	0.24; ±1.44	0.18; ±1.12	.316
**Offensive Individual Tactical-Related Variables**						
Total Number of Completed Passes (n)	4.90±2.79 (81.9%)	4.33±2.69 (83.9%)	4.54±2.60 (82.3%)	-0.56; ±0.76	-0.35; ±0.65	.513
Total Number of Failed Passes (n)	1.08±1.11 (18.1%)	0.85±1.00 (16.5%)	0.98±1.10 (17.7%)	-0.2; ±0.3	-0.1; ±0.4	.536
Total Number of Forward Passes (n)	1.35±1.41 (22.6%)	1.19±1.50 (23%)	1.06±1.42 (19.2%)	-0.17; ±0.30	-0.29; ±0.38	.082
Total Number of Lateral Passes (n)	2.65±2.10 (44.3%)	2.13±2.04 (41.1%)	2.54±1.99 (46.0%)	-0.52; ±0.62	-0.10; ±0.58	.676
Total Number of Backward Passes (n)	0.90±1.12 (15.0%)	1.02±1.00 (19.8%)	0.94±0.98 (17%)	0.13; ±0.29	0.04; ±0.36	.907
Total Number of Dribbles (n)	5.44±2.86	5.35±3.08	5.21±3.08	-0.08; ±0.82	-0.23; ±0.81	.698
Total Distance Covered during the Dribble (m)	34.11±23.64	36.67±27.81	37.32±29.26	2.56; ±6.08	3.21; ±7.58	.939
Total Number of Shots (n)	0.46±0.62	0.21±0.41	0.23±0.52	-0.25; ±0.16	-0.23; ±0.15	**.002**
Average Distance to Nearest Defender during the Shot (m)	1.33±1.99	0.53±1.29	1.73±8.4	-0.8±0.52; ±7.09	-0.4; ±2.03	**.007**
**Offensive Collective Tactical-Related Variables**						
Longitudinal Synchronization (%)	64.26±13.71	62.1±12.05	60.08±15.86	-2.16; ±2.56	-4.18; ±3.17	.065
Lateral Synchronization (%)	45.13±15.73	46.01±13.89	44.58±15.59	0.87; ±2.26	-0.56; ±2.45	.544
Distance to Nearest Teammate (m)	7.40±1.09	7.70±1.47	7.65±1.34	0.30; ±0.23	0.25; ±0.22	**.050**
Distance to Nearest Teammate (CV)	39.96±4.67	40.52±5.46	37.8±6.38	0.57; ±1.69	-2.16; ±1.73	.066
Distance to Nearest Opponent (m)	5.14±0.90	5.31±1.17	5.25±1.14	0.17; ±0.23	0.12; ±0.24	.472
Distance to Nearest Opponent (CV)	52.55±7.3	50.8±7.38	51.38±8.28	-1.75; ±2.32	-1.17; ±2.27	.460
Length (m)	18.93±3.49	17.64±2.24	20.57±4.86	-1.3; ±1.66	1.64; ±1.44	.067
Width (m)	21.47±1.85	22.42±2.83	22.73±3.03	0.95; ±1.24	1.25; ±1.47	.260

**Note**: CV, Coefficient of variation.

### Defensive phase

**Fig 2:**
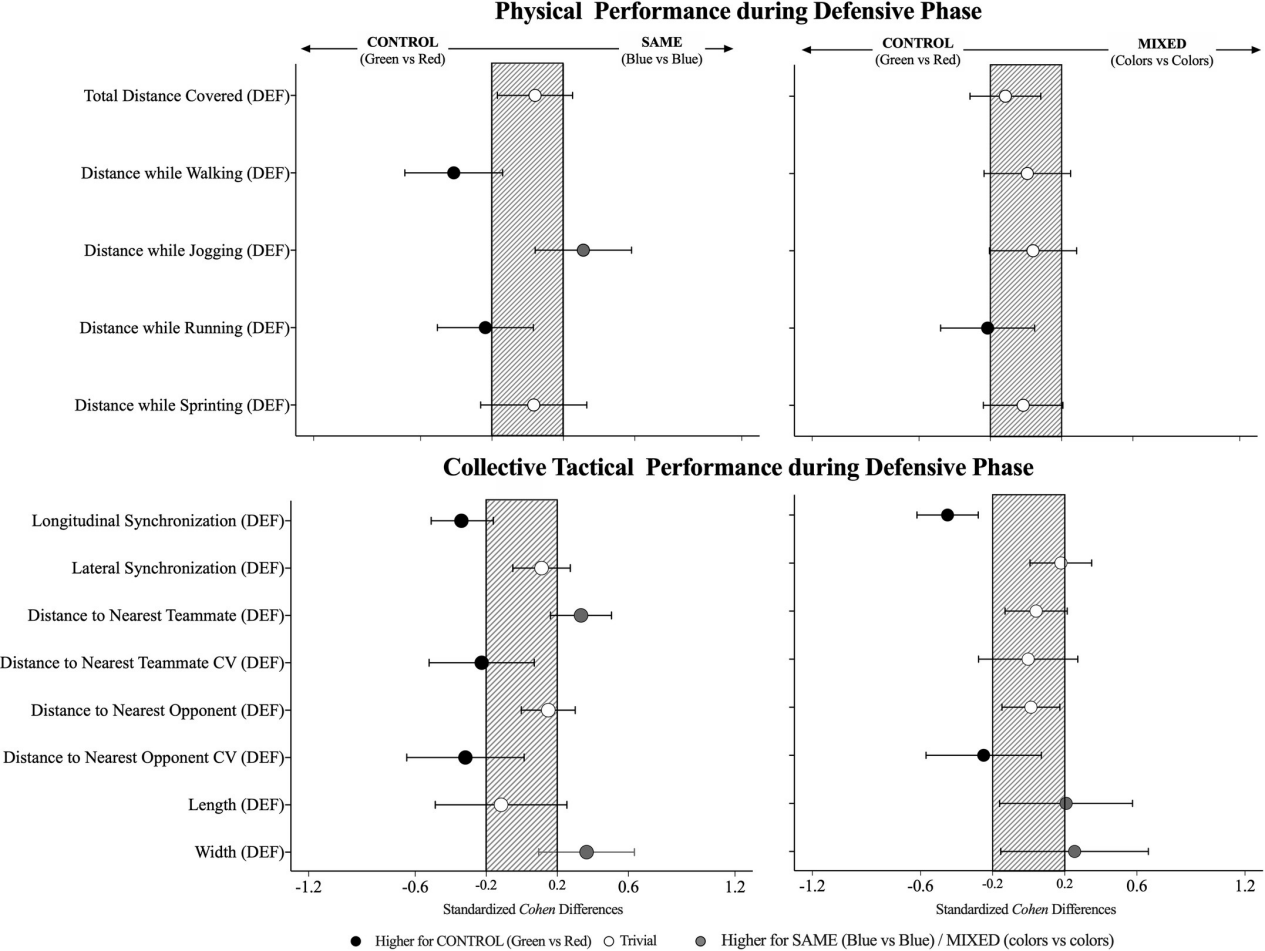
Standardised (Cohen) differences in physical and collective tactical variables according to the type of vest (CONTROL, SAME and MIXED) during the defensive phase. Error bars indicate uncertainty in the true mean changes with 90% confidence intervals. DEF = defensive.

The effects on the physical and collective tactical variables when manipulating the vests during the defensive phase are presented in Table 2, and Fig 2. From the physical perspective, some trends can be identified like the higher values of distance covered while walking and running in the CONTROL compared to the SAME. In contrast, there were lower values in the distance covered while jogging during the CONTROL compared to the SAME (small effects). When defending, the results from the collective tactical performance showed small higher values in longitudinal synchronization during the CONTROL compared to the SAME and MIXED (-4.92; ±2.56; and -6.56; ±2.53 respectively, X^2^ = 9.22, *P* **≤** 0.05). Also, it was identified a small lower distance between teammates when comparing the CONTROL with the SAME condition (0.52; ±0.27; *F* = 5.83, *P* **≤** 0.05). In addition, there were higher values in the CV from the distance to the nearest teammate in the CONTROL compared to the SAME (small effects), and higher values in the CV from the distance to the nearest opponent when comparing the CONTROL to both conditions (small effects). Finally, the results also showed lower values for the length during the CONTROL compared to the MIXED (~0.9 less, small effects) and in the width when comparing the CONTROL to the SAME and MIXED respectively (~1.0 less, small effects).

**Table 2:** Descriptive and statistical analysis for physical and collective tactical-related variables according to the type of vest used during the defensive phase.

Variables	Game-Based Conditions			Difference in means (±90% CL)		
	CONTROL	SAME	MIXED	CONTROL vs SAME	CONTROL vs MIXED	*P*
	(Green vs Red)	(Blue vs Blue)	(Colours vs Colours)			
	(Mean±SD)	(Mean±SD)	(Mean±SD)			
**Defensive Physical Variables**						
Total Distance Covered (m)	125.31±15.6	126.06±19.34	123.22±18.09	0.75; ±3.82	-2.09; ±3.60	.404
Distance Covered while Walking (m)	7.33±1.98	6.35±2.51	7.35±2.42	-0.98; ±0.65	0.02; ±0.57	.071
Distance Covered while Jogging (m)	91.41±12.4	95.72±13.38	91.96±14.6	4.3; ±3.73	0.55; ±3.38	.269
Distance Covered while Running (m)	21.76±9.4	19.2±11.52	19.44±10.58	-2.56; ±2.9	-2.32; ±2.84	.360
Distance Covered while Sprinting (m)	4.56±6.33	4.77±6.99	4.46±4.66	0.21; ±1.84	-0.1; ±1.39	.500
**Defensive Collective Tactical-Related Variables**						
Longitudinal Synchronization (%)	64.93±12.51	60.01±14.7	58.38±15.86	-4.92; ±2.56	-6.56; ±2.53	**.010**
Lateral Synchronization (%)	36.50±12.93	38.11±14.49	39.08±15.36	1.61; ±2.33	2.57; ±2.47	.196
Distance to Nearest Teammate (m)	8.23±1.51	8.75±1.52	8.29±1.57	0.52; ±0.27	0.06; ±0.27	**.004**
Distance to Nearest Teammate (CV)	40.68±5.09	39.26±5.88	40.66±7.32	-1.42; ±1.87	-0.02; ±1.73	.248
Distance to Nearest Opponent (m)	5.58±1.44	5.80±1.53	5.60±1.48	0.23; ±0.23	0.02; ±0.24	.570
Distance to Nearest Opponent (CV)	54.57±7.93	52.12±7.16	52.64±7.59	-2.45; ±2.55	-1.93; ±2.47	.594
Length (m)	20.32±2.68	19.82±3.77	21.22±4.25	-0.5; ±1.6	0.90; ±1.59	.417
Width (m)	24.29±2.51	25.44±2.61	25.09±2.82	1.15; ±0.85	0.80; ±1.29	.222

**Note**: Coefficient of variation; CL, Confidence limits.

## Discussion

This study explored how the players’ vests colour affects physical, individual, and collective tactical performance during a Gk+6vs6+Gk MSG. Previous research, also focused on changing the perceptual demands of players, has shown adaptative movement behaviour as a result of variations in the number of targets and its location [38, 39], as well as result of manipulations in the spatial references [2, 3]. In general, there were small differences between the conditions manipulated in the current study. From this perspective, results also suggest that players may rely their decisions also on other sources of information. In fact, a recent study explored the player’s sources of information during a 11vs11 match, and identified the ball, the opponent, the team and the player in possession as main sources of fixation locations [40]. In this respect, also the body position of players (i.e., offensive or defensive profile) may also provide information that may assist them in identifying the surrounding players as being teammates or opponents. Altogether, while the players’ vests may enhance or impair their ability to identify the teammates, it seems to be part of the sources of information in which they support their decisions, resulting in the small number of differences identified between the conditions. Nevertheless, the results from this study showed that playing with different vests (CONTROL) emphasized the offensive performance as players shown higher longitudinal synchronization (CONTROL VS SAME), a greater number of shots and also had, in general, higher values of forward passes. In contrast, during the SAME and MIXED scenarios, players explored the available space, mainly the width, possibly as an attempt to receive the ball with more time to act. In addition, the increased perceptual demands resulting from playing with the same vests (SAME) or several colour vests (MIXED) may limit the identification of forward passing lines. As players defensive behaviour seems to be dependent upon the teammates and opposing team positioning, could be the explanation for the higher movement synchronization found during the CONTROL while defending, as well as a higher focus on the lateral synchronization during the SAME and MIXED scenarios as result of the higher use of the field width. When considering the offensive and defensive phase, higher effects were found while defending. These results may be linked with a higher emphasis in coordinating the actions with the teammates to restrain and limit the available space while attempting to identify the offensive movements by the opposing team. By playing under the SAME or the MIXED condition, it may be possibly that players were less capable to establish these interactions with teammates and opponents contributing to such effects.

### Offensive phase

Varying the colour vests seems to have an impact on the players’ performance, mainly in the individual and collective behaviour. The players’ performance in association football is dependent upon their ability to interact with the surrounding information [1, 7, 41]. However, during the SAME / MIXED conditions, players wear vests that are likely to increase the perceptual demands. Consequently, these conditions affect the way how players use the environmental information, leading to adaptative movement behaviours. In fact, it was possible to identify a higher distance travelled with the ball in these two conditions. These results seem to suggest that players may face more difficulties in identifying teammates and opponents due to the increased perceptual demands. In fact, players have to move to perceive and interact with the environment [7], however these perceptual demands seems to increase when playing with the same colour vests or with several colours per team, forcing the players to stay more time on the ball and to use safer passing options (i.e., backward passes). In addition, the higher use of width in both the SAME and MIXED conditions compared to the CONTROL, as well as the higher distance to the nearest teammate (SAME) and length (MIXED) seems to support these trends. That is, players under such scenarios seem to explore more the available space on the field in an attempt to receive the ball with more space and time to act [42], as a result of the increased perceptual demands while the use of different vests (CONTROL) amplified the information of teammates and opponents positions and allow the ball carrier to easily sustain their actions.

In contrast, during the CONTROL each team wore a different colour vest (Green vs Red), which makes it easier to identify available passing options (mainly forward ones), and may justify the increase in the number of completed shots as it may allow to progress towards the opponents’ target. In fact, players’ performance is supported by visual information retrieved from the competitive performance environment [41, 43], such as teammates and opponents positioning [40]. Also, as a result of being easier to identify players belonging to each team, they do not need to stand so far apart in the field, allowing to stay more compact compared to the other two conditions. That is, while using all the available space is an offensive principle, players may also to maintain a certain distance between them to ensure that if a ball is lost they are able to proper press the opponents and protect their goal. In contrast, as result of being more difficult to identify the teammates under the SAME and MIXED condition, it is possibly that players increase their distance to have more time to perceive the environment, and affected their movement synchronization. In fact, the spatial proximity seems to favour the team movement synchronization, as players are closer to each other [2, 3, 34], which may support the higher values identified in the CONTROL condition. In addition, it seems reasonable to identify lower values in the movement synchronization during the SAME / MIXED conditions, as players may not be able to move together if they cannot properly identify the teammates.

However, to cope with the higher compactness, players may have to vary more often their distance with teammates. In fact, the results shown a higher coefficient of variation in the distance to the teammates and opponents found during the CONTROL. Accordingly, increasing the variability in the players’ behaviour, such as in the distance between players, has been found to be fundamental to face the perturbations that emerge from dynamic competitive environments [13].

The effects of varying the type of vests colours had minimal effects on the players’ physical performance while in possession. Nevertheless, players covered less total distance in the CONTROL than in the SAME, which may be possibly linked with the higher number of lateral and backward passes, which is likely to make the players cover more distance to provide passing lines and maintain the ball possession.

### Defensive phase

The players’ behaviour on the defensive phase was related to the offensive behaviour of the opposing team [44, 45]. That is, despite the team game model and strategy, it is somehow expected that the defensive team increases the time on the wide channels when the opposing team attempts to create overloads in the area, while in turn, add more players in the central channel to limit the opportunities for action in a team that explore the central zones (e.g., progressive possession style of play), which may lead to a higher longitudinal synchronization. Based on these assumptions, and taking into consideration the results found during the offensive phase, it is possible to understand the results observed during the defensive phase. That is, while attacking when playing in the CONTROL condition, players revealed a higher number of forward passes, number of shots and a higher longitudinal synchronization. These results suggest that use of the field longitudinal direction as the major direction of play while attacking [6, 36], which may also justify the increase in the longitudinal synchronization while defending, as an attempt from the team without possession to prevent the opponent to progress in the field and protect the goal. These results may also justify the higher distance covered while running in this condition comparing to the SAME and MIXED, as players not only use more the field length but also shot from a higher distance. In this regard, research surrounding the attacker-defender coordination tendencies have shown that the attacker attempts to increase the distance to the defender to score [44], while in turn the defender should maintain its position between the player in possession, the ball and the goal. This behaviour may force the defensive player to run in order to press the player in possession trying to avoid possible shooting opportunities. In fact, the higher variability in the distance to both teammates and opponents found during the CONTROL support this evidence.

In contrast, there was a higher focus on playing with field width during the SAME and MIXED scenarios, possibly as an attempt to receive the ball with more space that allow players to make better decisions. So, general higher values found in the lateral synchronization for these two conditions seems to be linked to the higher use of field lateral spaces by the offensive team, that consequently led the defensive team to occupy these spaces to press the player in possession and avoid possible goal scoring opportunities.

Whilst this study adds novel findings regarding to the effects of the varying the colour of the vests during MSG, some limitations should acknowledge. Since players’ performance results from the interaction of the individual, task and environmental constraints, it is likely that players from different expertise levels interact differently with the environmental information, and so it is possible that more expert players exhibit distinct behaviours due to their higher ability to perceive the relevant information. However, further research is needed to confirm this hypothesis. To accomplish such goals, it would be important to assess the players’ perceptual abilities and based on the results create different categories (i.e., low abilities, medium abilities, and high abilities) to understand whether this manipulation impact players with different ability to interact with the environment under different time-periods (i.e., short, mid and long-term). Similarly, the lower magnitude of effects found may suggest that apart from the colour equipment (vests), players may also rely in other sources of information, and so, further research is required to better understand which information guides their behaviour. In addition, it is important to note that players previous experiences seems to affects their ability to perform and adapt during competitive performances [46], and so, further studies should consider also exploring how different development paths affects the players’ perception. Furthermore, the goalkeeper has become an important part in the modern association football offensive patterns, where the change in the rules increased his participation. Taking this perspective into consideration, it would be important to consider in further studies the goalkeeper positioning and actions as his behaviour (i.e., a goalkeeper technically developed may be used as an extra offensive player during the build-up phase, or in turn, a less developed goalkeeper may inhibit his teammates to pass to him when under pressure and adopt longer passes) may impact players’ positioning.

### Practical applications

Overall, the results from this study showed how players adapted their movement behaviours as a result of variations in the colour of the players vests during MSG. For example, coaches may use different vests (CONTROL condition) to emphasize offensive behaviour as there were higher longitudinal synchronization, forward passes and shots on target. Similarly, this condition can be used to emphasize defensive behaviours related to the field longitudinal direction (e.g., train players depth control while defending) as players were more synchronized while defending in this condition. In turn, attacking while both teams using the same vests (SAME) or using several colours (MIXED) seems to emphasize the use of field available space as an attempt of the players to receive the ball further from opponents to have more time and space to act. For that purpose, players used the lateral spaces, contributing to higher movement coordination in the lateral direction, which can be used by coaches to emphasize the behaviour of change the point-of-attack. Despite not being a condition faced during competitive matches (i.e., players from both teams wear distinct outfits), it may be used by the coaches to emphasize specific behaviours such as space exploration while amplifying the perceptual demands. In fact, this have been a common strategy during training sessions (e.g., limiting the number of allowed touches), where coaches create more complex scenarios during sessions that allow the players to face the game with more confidence.

## Conclusions

Overall, the results from this study reveal how players adjust their movement in defence and attack as result of changes in the colours of players vests. Differences between conditions were observed particularly in individual and collective tactical related variables. Since players behaviour is dependent upon their ability to use the environmental information, coaches may vary the type of vests used to amplify the need to interact with the surrounding competitive environment.

## References

[1] Travassos B, Araújo D, Davids K, O’Hara K, Leitão J, Cortinhas A. Expertise effects on decision-making in sport are constrained by requisite response behaviours–A meta-analysis. Psychology of Sport and Exercise. 2013;14(2):211–9.

[2] Coutinho D, Gonçalves B, Travassos B, Folgado H, Figueira B, Sampaio J. Different Marks in the Pitch Constraint Youth Players’ Performances During Football Small-sided Games. Res Q Exerc Sport. 2020;91(1):15–23. doi: 10.1080/02701367.2019.1645938 31479411

[3] Coutinho D, Gonçalves B, Travassos B, Abade E, Wong DP, Sampaio J. Effects of pitch spatial references on players’ positioning and physical performances during football small-sided games. Journal of Sports Sciences. 2018:1–7. doi: 10.1080/02640414.2018.1523671 30306840

[4] Gonçalves B, Marcelino R, Torres-Ronda L, Torrents C, Sampaio J. Effects of emphasising opposition and cooperation on collective movement behaviour during football small-sided games. J Sports Sci. 2016;34(14):1346–54. doi: 10.1080/02640414.2016.1143111 26928336

[5] Gonçalves B, Coutinho D, Exel J, Travassos B, Lago C, Sampaio J. Extracting spatial-temporal features that describe a team match demands when considering the effects of the quality of opposition in elite football. Plos One. 2019;14(8):e0221368. doi: 10.1371/journal.pone.0221368 31437220 PMC6705862

[6] Folgado H, Bravo J, Pereira P, Sampaio J. Towards the use of multidimensional performance indicators in football small-sided games: the effects of pitch orientation. J Sports Sci. 2019;37(9):1064–71. doi: 10.1080/02640414.2018.1543834 30426856

[7] Gibson JJ. The Ecological Approach to Visual Perception: Lawrence Erlbaum Associates; 1986.

[8] Travassos B, Duarte R, Vilar L, Davids K, Araujo D. Practice task design in team sports: representativeness enhanced by increasing opportunities for action. J Sports Sci. 2012;30(13):1447–54. doi: 10.1080/02640414.2012.712716 22871067

[9] Fajen B, Riley M, Turvey M. Information, Affordances, and the Control of Action in Sport. International Journal of Sport Psychology. 2009(40):79–107.

[10] Pinder RA, Davids K, Renshaw I, Araujo D. Representative learning design and functionality of research and practice in sport. Journal of Sport and Exercise Psychology. 2011;33(1):146–55. doi: 10.1123/jsep.33.1.146 21451175

[11] Davids K, Araújo D, Correia V, Vilar L. How small-sided and conditioned games enhance acquisition of movement and decision-making skills. Exercise and sport sciences reviews. 2013;41(3):154–61. doi: 10.1097/JES.0b013e318292f3ec 23558693

[12] Davids K, Chow J, Shuttleworth R. A constraints-based framework for nonlinear pedagogy in physical education. Journal of Physical Education New Zealand. 2005;38(1):17–29.

[13] Seifert L, Button C, Davids K. Key properties of expert movement systems in sport: an ecological dynamics perspective. Sports Medicine. 2013;43(3):167–78. doi: 10.1007/s40279-012-0011-z 23329604

[14] Santos S, Coutinho D, Gonçalves B, Schöllhorn W, Sampaio J, Leite N. Differential Learning as a Key Training Approach to Improve Creative and Tactical Behavior in Soccer. Research Quarterly for Exercise and Sport. 2018;89(1):11–24. doi: 10.1080/02701367.2017.1412063 29351500

[15] Coutinho D, Gonçalves B, Travassos B, Abade E, Wong DP, Sampaio J. Effects of pitch spatial references on players’ positioning and physical performances during football small-sided games. Journal of Sports Sciences. 2019;37(7):741–47. doi: 10.1080/02640414.2018.1523671 30306840

[16] Gonçalves B, Esteves P, Folgado H, Ric A, Torrents C, Sampaio J. Effects of pitch area-restrictions on tactical behavior, physical and physiological performances in soccer large-sided games. J Strength Cond Res. 2016;Advance Online Publication. 10.1519/JSC.0000000000001700 27806007

[17] Low B, Coutinho D, Gonçalves B, Rein R, Memmert D, Sampaio J. A Systematic Review of Collective Tactical Behaviours in Football Using Positional Data. Sports Medicine. 2020;50(2):343–85. doi: 10.1007/s40279-019-01194-7 31571155

[18] Machado JC, Barreira D, Teoldo I, Travassos B, Júnior JB, Santos JOLD, et al. How Does the Adjustment of Training Task Difficulty Level Influence Tactical Behavior in Soccer? Research quarterly for exercise and sport. 2019;90(3):403–16. doi: 10.1080/02701367.2019.1612511 31157599

[19] Natsuhara T, Kato T, Nakayama M, Yoshida T, Sasaki R, Matsutake T, et al. Decision-Making While Passing and Visual Search Strategy During Ball Receiving in Team Sport Play. Perceptual and Motor Skills. 2020;127(2):468–89. doi: 10.1177/0031512519900057 31964223

[20] Vaeyens R, Lenoir M, Williams AM, Mazyn L, Philippaerts RM. The effects of task constraints on visual search behavior and decision-making skill in youth soccer players. Journal of Sport and Exercise Psychology. 2007;29(2):147–69. doi: 10.1123/jsep.29.2.147 17568064

[21] McGuckian TB, Cole MH, Jordet G, Chalkley D, Pepping G-J. Don’t Turn Blind! The Relationship Between Exploration Before Ball Possession and On-Ball Performance in Association Football. Frontiers in psychology. 2018;9:2520–. doi: 10.3389/fpsyg.2018.02520 30618946 PMC6295565

[22] McGuckian TB, Cole MH, Chalkley D, Jordet G, Pepping G-J. Constraints on visual exploration of youth football players during 11v11 match-play: The influence of playing role, pitch position and phase of play. Journal of Sports Sciences. 2020;38(6):658–68. doi: 10.1080/02640414.2020.1723375 32009533

[23] Jordet G, Bloomfield J, Heijmerikx J. The Hidden Foundation of Field Vision in English Premier League (Epl) Soccer Players. The Mit Sloan Sports Analytics Conference; Boston2013.

[24] Attrill MJ, Gresty KA, Hill RA, Barton RA. Red shirt colour is associated with long-term team success in English football. J Sports Sci. 2008;26(6):577–82. doi: 10.1080/02640410701736244 18344128

[25] Olde Rikkert J, Haes VD, Barsingerhorn AD, Theelen T, Olde Rikkert MG. The colour of a football outfit affects visibility and team success. J Sports Sci. 2015;33(20):2166–72. doi: 10.1080/02640414.2015.1064156 26140538

[26] Balakrishnan G, Uppinakudru G, Girwar Singh G, Bangera S, Dutt Raghavendra A, Thangavel D. A Comparative Study on Visual Choice Reaction Time for Different Colors in Females. Neurology Research International. 2014;2014:301473. doi: 10.1155/2014/301473 25580294 PMC4280496

[27] Burnell L, Thompson P. Finding Neymar: The Role of Colour in the Detection and Discrimination of Football Kits. Perception. 2021;50(7):615–26. doi: 10.1177/03010066211019370 34107814 PMC8258731

[28] Hüttermann S, Smeeton NJ, Ford PR, Williams AM. Color Perception and Attentional Load in Dynamic, Time-Constrained Environments. Frontiers in psychology. 2019;9:2614–. doi: 10.3389/fpsyg.2018.02614 30670996 PMC6331534

[29] Faul F, Erdfelder E, Lang AG, Buchner A. G* Power 3: A flexible statistical power analysis program for the social, behavioral, and biomedical sciences. Behavior research methods. 2007. 10.3758/bf03193146 17695343

[30] Casamichana D, Castellano J. Time-motion, heart rate, perceptual and motor behaviour demands in small-sides soccer games: effects of pitch size. J Sports Sci. 2010;28(14):1615–23. doi: 10.1080/02640414.2010.521168 21077005

[31] Silva P, Vilar L, Davids K, Araújo D, Garganta J. Sports teams as complex adaptive systems: manipulating player numbers shapes behaviours during football small-sided games. SpringerPlus. 2016;5(1):191. doi: 10.1186/s40064-016-1813-5 27026887 PMC4769238

[32] Barbero-Alvarez JC, Lopez MG, Castagna C, Barbero-Alvarez V, Romero DV, Blanchfield AW, et al. Game Demands of 7-a-Side Soccer in Young Players. J Strength Cond Res. 2015. 10.1519/JSC.0000000000001143 26349040

[33] Aguiar M, Goncalves B, Botelho G, Lemmink K, Sampaio J. Footballers’ movement behaviour during 2-, 3-, 4- and 5-a-side small-sided games. J Sports Sci. 2015;33(12):1259–66. doi: 10.1080/02640414.2015.1022571 25782702

[34] Folgado H, Duarte R, Fernandes O, Sampaio J. Competing with lower level opponents decreases intra-team movement synchronization and time-motion demands during pre-season soccer matches. Plos One. 2014;9(5):e97145. doi: 10.1371/journal.pone.0097145 24817186 PMC4016249

[35] O’Donoghue P. Research methods for sports performance analysis. London: Routledge; 2010. 278 p.

[36] Folgado H, Duarte R, Marques P, Sampaio J. The effects of congested fixtures period on tactical and physical performance in elite football. J Sports Sci. 2015;33(12):1238–47. doi: 10.1080/02640414.2015.1022576 25765524

[37] Hopkins WG, Marshall SW, Batterham AM, Hanin J. Progressive statistics for studies in sports medicine and exercise science. Medicine & Science in Sports & Exercise. 2009;41(1):3–13. doi: 10.1249/MSS.0b013e31818cb278 19092709

[38] Travassos B, Coutinho D, Gonçalves B, Pedroso P, Sampaio J. Effects of manipulating the number of targets in U9, U11, U15 and U17 futsal players’ tactical behaviour. Hum Mov Sci. 2018;61:19–26. doi: 10.1016/j.humov.2018.06.017 30005844

[39] Coutinho D, Gonçalves B, Santos S, Travassos B, Wong DP, Sampaio J. Effects of the pitch configuration design on players’ physical performance and movement behaviour during soccer small-sided games. Research in Sports Medicine. 2019;27(3):298–313. doi: 10.1080/15438627.2018.1544133 30394800

[40] Aksum K, Magnaguagno L, Bjørndal C, Jordet G. What Do Football Players Look at? An Eye-Tracking Analysis of the Visual Fixations of Players in 11 v 11 Elite Football Match Play. Frontiers in psychology. 2020;11(2624). 10.3389/fpsyg.2020.562995 PMC7596273 33178070

[41] Faber LG, Maurits NM, Lorist MM. Mental fatigue affects visual selective attention. Plos One. 2012;7(10):e48073. doi: 10.1371/journal.pone.0048073 23118927 PMC3485293

[42] Brito R, Bredt S, Greco J, Clemente F, Teoldo I, Praça G. Influence of limiting the number of ball touches on players’ tactical behaviour and network properties during football small-sided games. International Journal of Performance Analysis in Sport. 2019;19(6):999–1010.

[43] Fajen BR. Perceiving possibilities for action: on the necessity of calibration and perceptual learning for the visual guidance of action. Perception. 2005;34(6):717–40. doi: 10.1068/p5405 16042193

[44] Vilar L, Araujo D, Travassos B, Davids K. Coordination tendencies are shaped by attacker and defender interactions with the goal and the ball in futsal. Human Movement Science. 2014;33:14–24. doi: 10.1016/j.humov.2013.08.012 24576704

[45] Duarte R, Araújo D, Correia V, Davids K, Marques P, Richardson MJ. Competing together: Assessing the dynamics of team-team and player-team synchrony in professional association football. Human Movement Science. 2013;32(4):555–66. doi: 10.1016/j.humov.2013.01.011 24054894

[46] Santos S, Mateus N, Sampaio J, Leite N. Do previous sports experiences influence the effect of an enrichment programme in basketball skills? Journal of Sports Sciences. 2016:1–9. doi: 10.1080/02640414.2016.1236206 27681710

